# Evaluation of different spectral indices for wheat lodging assessment using machine learning algorithms

**DOI:** 10.1038/s41598-025-09109-5

**Published:** 2025-07-01

**Authors:** Shikha Sharda, Sumit Kumar, Raj Setia, Prince Dhiman, N. R. Patel, Brijendra Pateriya, Ali Salem, Ahmed Elbeltagi

**Affiliations:** 1 Punjab Remote Sensing Centre, Ludhiana, Punjab India; 2 Indian Institute of Remote Sensing, Indian Space Research Organisation, Dehradun, India; 3 Civil Engineering Department, Faculty of Engineering, Minia University, Minia, Egypt; 4 Structural Diagnostics and Analysis Research Group, Faculty of Engineering and Information Technology, University of Pecs, Pecs, Hungary; 5 Agricultural Engineering Department, Faculty of Agriculture, Mansoura University, Mansoura, 35516 Egypt

**Keywords:** Lodging, Machine learning, Sentinel-2, Wheat, Evolution, Plant sciences

## Abstract

Wheat lodging is a recurrent phenomenon that significantly affects grain yield and impedes the harvesting efficiency. Therefore, the precise and rapid assessment of wheat lodging is crucial in minimizing its impact on grain yield and quality. Recently few studies related to machine learning based wheat lodging have been reported; however, the literature still lacks comprehensive assessments of machine learning algorithms for wheat lodging over Indian agricultural fields. This study presented a systematic approach for detecting the wheat lodging occurred during the end of March and April 2023 in the Ludhiana district of Punjab (India) from multi-temporal Sentinel-2 data using the machine learning algorithms. The ground control points for healthy and lodged areas were collected during March and April 2023. The temporal characteristics of crop phenology from November 2022 to April 2023 were analyzed for wheat classification. The normalized difference vegetation index (NDVI) was computed during this period followed by implementation of random forest (RF), decision tree (DT), and support vector machine (SVM) algorithms to evaluate their performance for wheat classification. It was found that RF outperformed the other models in terms of prediction accuracy and wheat area extraction. To distinguish between lodged and non-lodged wheat, eight spectral indices were computed from the visible and infrared bands of Sentinel-2. These indices were used as inputs to RF, DT, and SVM models. The optimal set of features were identified using random forest feature importance selection approach. Among the spectral indices, spectral sum index (SSI) derived from blue, green, red, and near-infrared bands followed by generalized difference vegetation index (GDVI) accurately separated lodged wheat from non-lodged wheat. Among the three algorithms, the RF model combined with SSI and GDVI achieved the highest overall accuracy of 89.2%. These results suggested that SSI and GDVI derived from Sentinel-2 data coupled with random forest model is effective for assessing the wheat lodging on spatio-temporal scale which may be helpful for developing the decision support system to assess the loss of crop yield loss.

## Introduction

Wheat is one of the major winter cereal crops of India and contributes almost one-third to the total food grain production in India^1^. Department Agriculture & Farmers Welfare^2^ estimated total wheat production of 110.55 million tons in India during the year 2022–2023. Climate variability and weather extremes such as heavy rain and strong winds significantly hinder the crop production and lead to lodging^3^. Crop lodging is characterized by the oblique and horizontal orientation of crop stalks, and this displacement affects grain yield and quality^4^. Therefore, accurate and timely estimate of the spatio-temporal information about wheat lodging is beneficial in minimizing yield loss^5^.

The traditional field survey method that utilizes visual inspection, is the widely used approach for the assessment of lodging, but it is laborious for large areas and subject to expert’s skill and knowledge^6^. Satellite-based remote sensing, capable of providing multi-temporal data with large-spatial coverage, provides a cost-effective alternative for crop lodging mapping^7^. With the advancement in remote sensing technology, several studies have utilized information from various sensors i.e., visible light sensors^8,9^, infrared sensors^10^, and microwave sensors^7,11^ to detect the crop lodging. A multi-temporal radar satellite data was utilized for assessing wheat lodging^7^. A high resolution unmanned aerial vehicle (UAV) data was used to examine the spectral variability between different lodging severity classes and showed that the red edge and near infrared (NIR) bands clearly separated lodged from non-lodged wheat with 90% accuracy^12^. Later,  Chauhan et al. ^3^ analyzed the capability of time-series SAR and optical data in extracting wheat lodging and distinguishing lodging severity. The results showed that red edge and NIR bands of Sentinel-2, and VH, VV and VH/VV backscatter extracted from Sentinel-1, best differentiated the healthy wheat from lodged wheat. Further, the lodged area was effectively mapped based on the temperature difference information between lodged and non-lodged wheat, finding it to be more precise than other methods such as color features, supervised classification, and canopy plant height model^10^. Though the existing studies show good performance in mapping the lodged fields, but few studies have used the satellite data and machine learning algorithms for rapid identification of crop lodging^8,13,14^. Machine learning algorithms coupled with the geospatial data offers a powerful way to analyze large datasets, automate tasks, and derive meaningful insights from earth observation data.

In recent years, machine learning-based algorithms gained widespread interest in precision agriculture and were introduced for automatic mapping of wheat lodging areas^15,16^. Zhao et al.^15^ investigated the potential of multi-growth stages UAV red–green–blue (RGB) images and a deep learning model for the automatic detection of wheat lodging and found 89.47% accurate. Zhang et al.^16^ compared three traditional machine learning algorithms, i.e., random forest (RF), neural network (NN), and support vector machine (SVM). They found that RF model combined with textural features identified lodged areas with 91% accuracy, which was the highest among other methods tested. The influence of adding digital surface model (DSM) and excess green index (ExG) features to UAV RGB image was studied for the identification of wheat lodged fields using deep learning models^8^. The experiment results showed that RGB and DSM recognized lodged field with a higher overall accuracy of 88.99%. A combination of various vegetation indices and edge features was utilized to train different deep learning-based models for extracting wheat lodging area^14^. Yu et al.^17^ combined the UAV RGB image with an improved Unet network and extracted wheat lodging with 88% accuracy. These studies show that available machine learning-based lodging assessment studies were restricted to field-specific applications due to small coverage of UAV imagery, and the implementation of the existing approaches over the large geographical areas remains a challenge. Furthermore, only limited research was conducted to study the influence of different spectral indices in extracting wheat lodged fields. Therefore, present study made an attempt to explore the potential of time-series multispectral satellite data combined with machine learning algorithms for the assessment of wheat lodging over large-scale Indian agricultural fields. The objectives of the present study were to: (1) evaluate the temporal characteristics of crop phenology based on NDVI computed during the crop growth stages for wheat classification, (2) analysis of different vegetation indices based on machine learning models for the assessment of wheat lodging, and (3) implement and evaluate the performance of traditional machine learning algorithms for wheat classification and extraction of wheat lodging areas. The proposed study was conducted in the wheat growing areas of Ludhiana district of Punjab, India during November 2022-April 2023. Three widely used state-of-art machine learning algorithms i.e., random forest (RF)^18^, decision tree (DT)^19^, and support vector machine (SVM)^18^, were implemented and their performance were compared for wheat classification and lodging detection.

## Study area

**Fig. 1:**
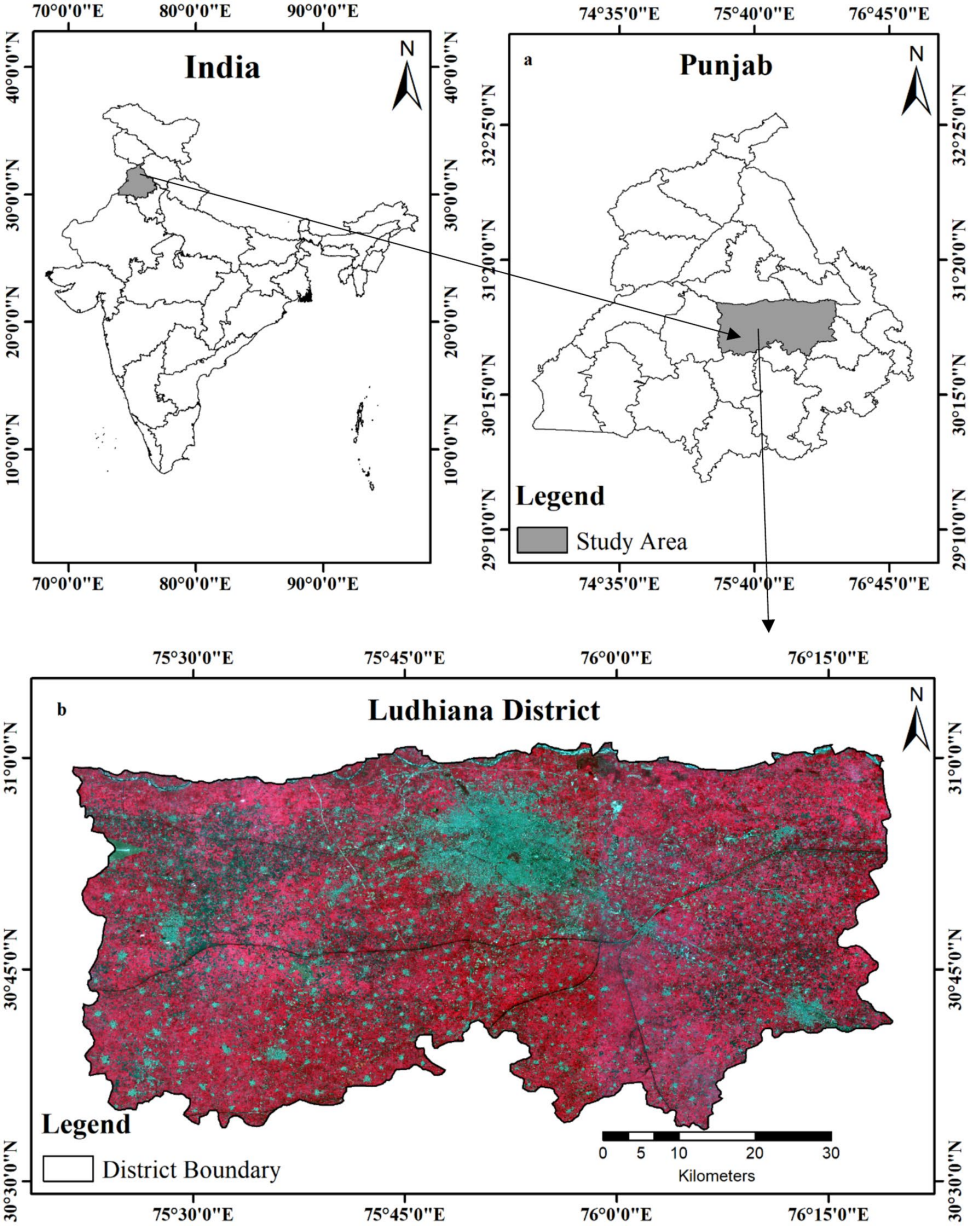
(**a**) Location of study area and (**b**) False-color composite (Bands: NIR/Red/Green) of Sentinel-2 image of experimental area for the assessment of wheat lodging.

The Ludhiana district is located in the central part of Punjab, India, with geographic coordinates of 30° 33ʹ–31° 01ʹ N and 75° 21ʹ–76° 19ʹ E (Fig. 1). The district is a part of Indo-Gangetic alluvial plain. The alluvial deposits belong to the Pleistocene to the recent times and vary in thickness. The mineralogical make-up of clay in the soils of the Ludhiana district shows that soils have moderate amounts of vermiculite and intergrade minerals (chloritized vermiculite and montmorillonite), feldspars and quartz, whereas chlorite usually does not occur in these soils. This suggests that mineral alteration or transformation from illite to vermiculite and further to chloritized vermiculite is active in these soils.

The climate of district is sub-tropical monsoonal climate, subject to south-easterly summer rains. The westerly atmospheric depressions cause winter rains. The total rainfall of the district averages 660 mm (average of 40 years). About 70% of the rainfall is received between July and September and coincides with most of the growth period of the summer crops. From October to the end of June, generally dry conditions prevail except for a few light showers received owing to westerly depressions. The district represents the extremes of climate. The summer temperatures are severely high, with May to June temperature ranging from 45 to 48 °C. The winters are fairly cold, with temperature touching very low on a few days during December to January. Frosts are fairly common during these months.

Wheat is the most widely cultivated crop in the district during the winter season. The crop is sown during early November and is harvested by the mid of April. At the end of March 2023, crop lodging in the district occurred due to strong winds and heavy rain . A field survey was conducted following the lodging event and a total of 277 ground control points covering both non-lodged and lodged crop areas across Ludhiana were collected during the first week of April 2023.

For time-series analysis, a set of six Sentinel-2 images was acquired over the selected study area during the wheat crop’s growth stage from November 2022 to April 2023 (Table 1). The Sentinel-2 images were downloaded with less than 10% cloud cover via the Google Earth Engine (GEE), a cloud-based platform.

**Table 1:** Phenology of wheat corresponding to the date of satellite data.

Satellite acquisition date	Crop growth stage
23 November 2022	Sowing
13 December 2022	Crown root initiation (CRI)
17 January 2023	Jointing
26 February 2023	Anthesis
23 March 2023	Grain filling
07 April 2023	Maturity

**Fig. 2:**
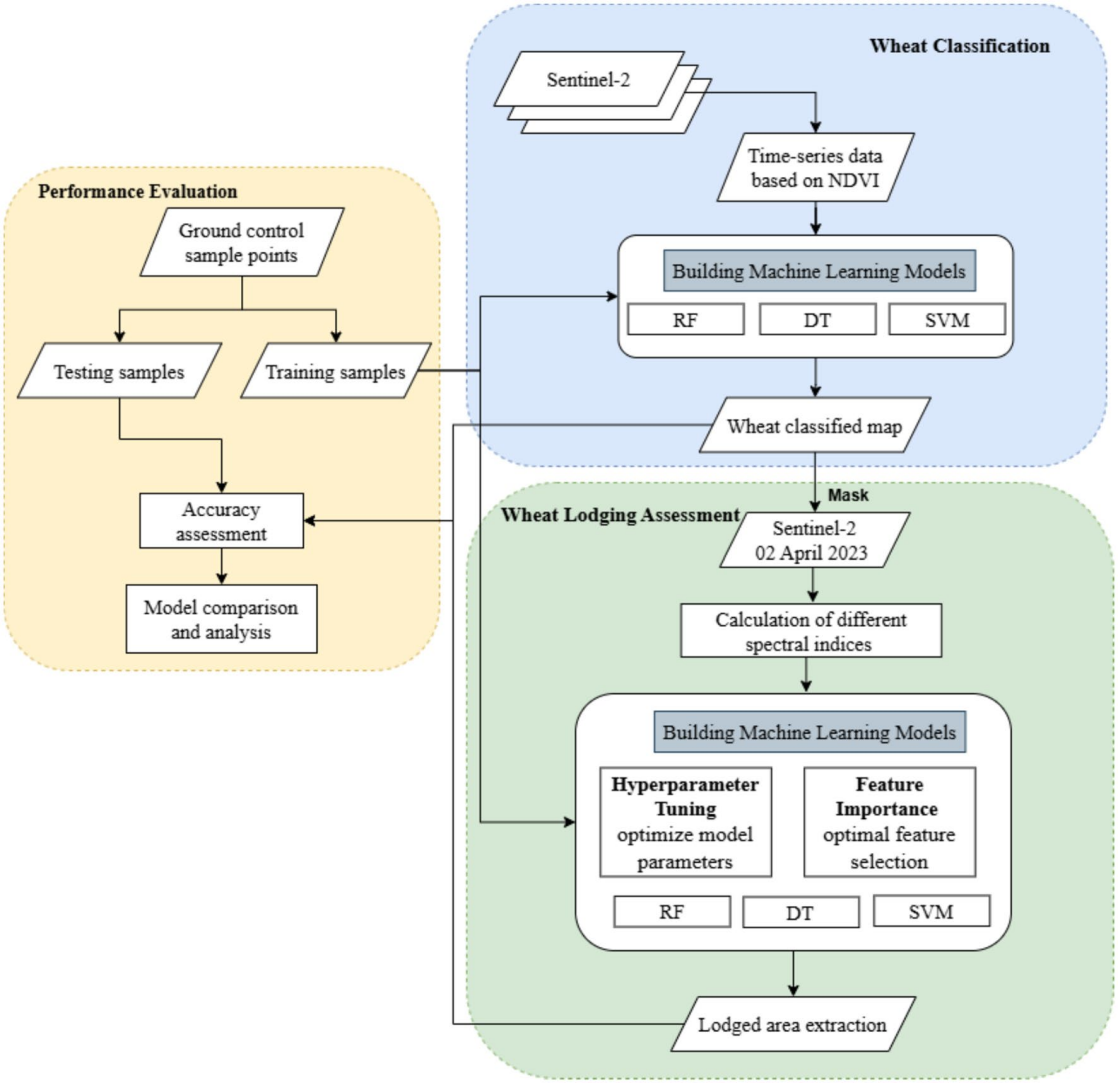
Flowchart of machine learning-based wheat lodging assessment.

The methodology of machine learning-based wheat lodging assessment in the Ludhiana district of Punjab is given in Fig. 2. The proposed approach comprises of the following steps: (1) Classification of wheat from multi-temporal Sentinel-2 data using machine learning algorithms, (2) Calculation of various vegetation indices and their performance analyses for lodged and non-lodged areas, (3) Optimization of machine learning model hyperparameters (Table 2) using Grid Search Cross-Validation (GridSearchCV) (4) Extraction of wheat lodging areas using the optimal set of features and machine learning algorithms, and (5) Performance evaluation of different ML models based on the confusion matrix statistical parameters.

**Table 2:** Hyperparameter tuning of machine learning algorithms.

Algorithms	Parameters	Range	Step	Description
RF	n_estimators	[100, 500]	50	The number of trees in the forest
	max_depth	[1, 300]	30	The maximum depth of the tree
	max_features	[8, 108]	10	Number of random features sampled per node
DT	criterion	[‘gini’, ‘entropy’]	–	Measure the quality of a split
	max_depth	[1, 300]	30	The maximum depth of the tree
SVM	C	[0.1, 1, 10, 100]	–	Regulation parameter
	kernal	[‘rbf’]	–	
	gamma	[0.001, 0.01, 0.1, 1]	–	Kernel coefficient

## Machine learning algorithms and the hypertuning of parameters

**Fig. 3:**
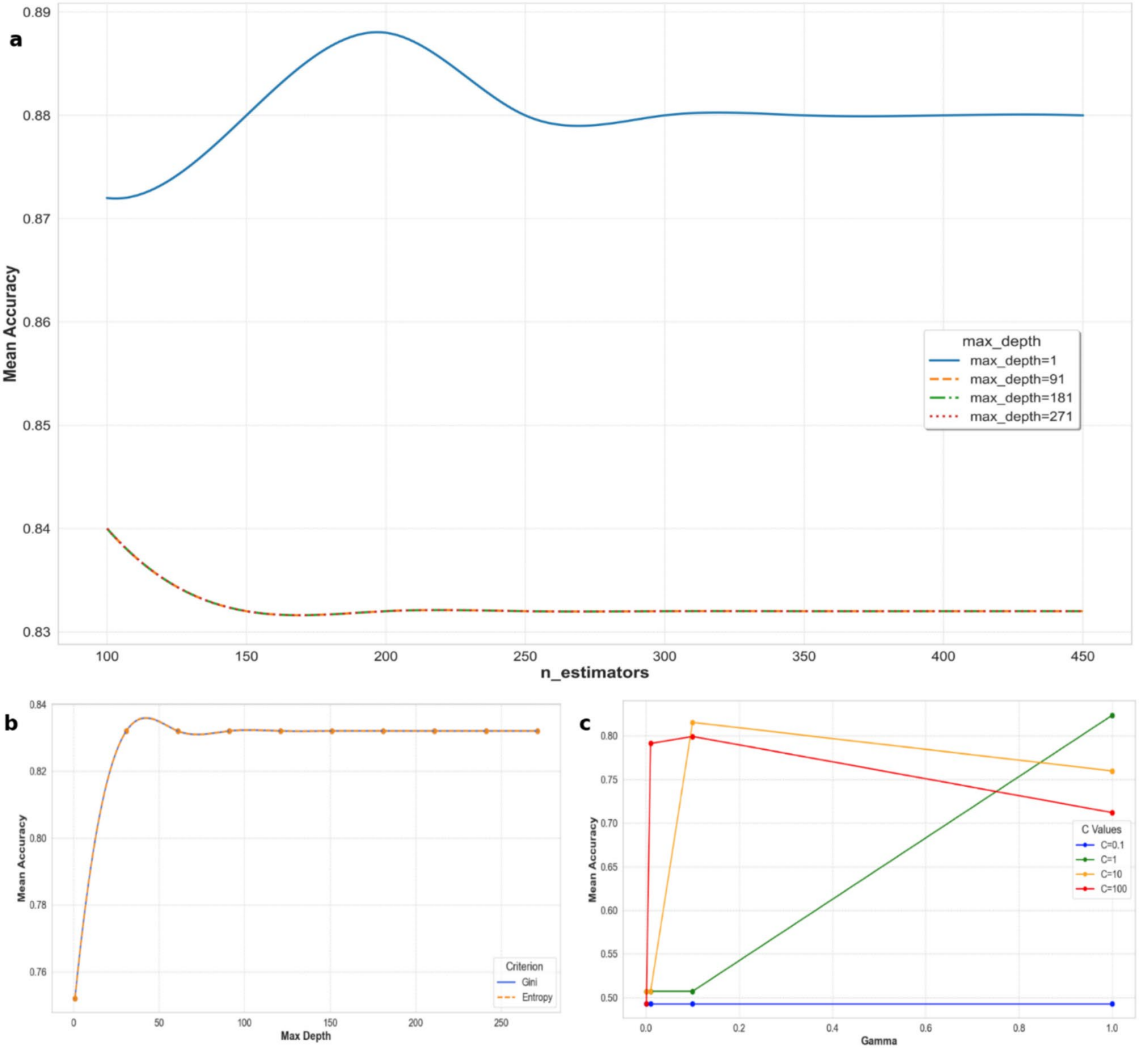
Performance of hyperparameters for (**a**) RF, (**b**) DT, and (**c**) SVM algorithms.

Three state-of-art machine learning algorithms namely random forest (RF), decision tree (DT), and support vector machine (SVM) were implemented in Python. Random forest is an ensemble supervised learning algorithm that integrates decision trees via boot strapping^18^. Unlike a single decision tree classifier, that include entire training dataset to create the decision rules, RF utilized a two-third of the training dataset to build multiple decision trees. The remaining one-third data is used to estimate misclassification error, referred as out-of-bag (OOB) error^20^. By aggregating the outputs of multiple trees, a random forest can provide more robust and reliable predictions for land-cover classification^21^. In the present study, three hyperparameters were tuned as follows: {‘n_estimators’: 200, ‘max_depth’: 1, ‘max_features’: 8} for building a model for the assessment of wheat lodging (Fig. 3).

Decision tree is a non-parametric supervised learning algorithm utilized for both classification and regression problems^22^. Among other DT algorithms (ID3, C4.5), Classification and regression tree (CART) is the most widely acceptable model for remote sensing applications. CART is capable in handling high-dimensional data and can be easily visualize and interpret^22^. CART forms a tree-structured framework by recursively partitions the data according to the defined classification framework. The two hyperparameters were set to: {‘criterion’: 'gini ', ‘max_depth’: 31} to build a model (Fig. 3).

Support vector machines are supervised statistical learning algorithms which have been vigorously explored in remote sensing for crop classification^23^. The SVMs algorithm determine an optimal hyper plane separating the dataset into predefined classes by using a non-linear kernel function. The commonly used kernel functions are grouped as linear, polynomial, radial basis function (RBF), and sigmoid kernels; however, RBF is widely used in the literature for land cover classification^23,24^. In the present study, the optimal hyperparameter values for building model were found to be {'C': 1, ‘kernel’: ‘rbf’, ‘gamma’: 1} (Fig. 3).

## Classification of wheat from Sentinel 2 data using machine learning algorithms

Time series NDVI data was generated by selecting cloud-free images from November 2022 to April 2023 to best capture the phenological characteristics of the crop^14,23,25^. NDVI values were calculated based on red and NIR bands of Sentinel-2 imageries, available at an interval of 5-days from November 2022 to April 2023. The ground samples (N = 277) were randomly partitioned into 60% training and 40% testing sets. NDVI time-series values were extracted for the training samples and created an excel datasheet file. The processed data was imported to train the machine learning models (RF, DT, and SVM).

## Wheat lodging assessment using spectral indices and machine learning algorithms

Table 3 shows the list of spectral indices for mapping crop lodged fields. The list of these indices are: ratio vegetation index (RVI), normalized difference vegetation index (NDVI), green normalized difference vegetation index (GNDVI), generalized difference vegetation index (GDVI), spectral sum index (SSI), enhanced vegetation index (EVI), soil adjusted vegetation index (SAVI), and excess green index (ExG). Prior to wheat lodging analysis, the Sentinel-2 image acquired on 2 April 2023 was masked using the extracted wheat layer. The spectral indices were derived from the visible and infrared bands of Sentinel-2 imagery acquired post lodging event. Subsequently, an analysis was conducted to study the spectral response of various indices for lodged and non-lodged wheat. The optimal features were identified using random forest feature importance selection (RF-FI) approach^26^, a statistical approach that ranked features based on importance scores. Further, these features were used to build the ML models. The performance of ML algorithms was quantitatively evaluated using the confusion matrix, and the classified results were analyzed using ground control sample points as a reference.

**Table 3:** Different spectral indices used in the present study.

Vegetation index	Formulae	References
Ratio vegetation index	$RVI=\frac{{\rho }_{NIR}}{{\rho }_{Red}}$	Tang et al.^14^
Normalized difference vegetation index	$NDVI= \frac{{\rho }_{NIR}-{\rho }_{Red}}{{\rho }_{NIR}+{\rho }_{Red}}$	Tang et al.^14, Kim et al.34^
Green difference vegetation index	$GNDVI= \frac{{\rho }_{NIR}-{\rho }_{Green}}{{\rho }_{NIR}+{\rho }_{Green}}$	Tang et al.^14, Kim et al.34^
Generalized difference vegetation index	$GDVI= {\rho }_{NIR}-{\rho }_{Green}$	Guan et al.^13^
Spectral sum index	$SSI= {\rho }_{Red}+{\rho }_{Green}+{\rho }_{Blue}+{\rho }_{NIR}$	Chen et al.^9^
Enhanced vegetation index	$EVI= 2.5 \times \frac{{\rho }_{NIR}-{\rho }_{Red}}{{\rho }_{NIR}+6\times {\rho }_{Red}-7.5\times {\rho }_{Blue}+1}$	Tang et al.^14^
Soil adjusted vegetation index	$SAVI=\frac{({\rho }_{NIR}-{\rho }_{Red})}{({\rho }_{NIR}+{\rho }_{Red}+L)}\times (1+L)$	Kim et al.^34^
Excess green index	$ExG=\frac{2\times {\rho }_{Green}-{\rho }_{Red}-{\rho }_{Blue}}{{\rho }_{Red}+{\rho }_{Green}+{\rho }_{Blue}}$	Liu et al.^33^, Yang et al.^8^

## Accuracy assessment

The performance evaluation of three classification models was evaluated based on the following statistical metrics ^27^:

1 $$Precision= \frac{TP}{(TP+FP)}$$

2 $$Recall= \frac{TP}{(TP+FN)}$$

3 $$F1-score= 2*\frac{Precision*Recall}{(Precision+Recall)}$$

4 $$Overall Accuracy= \frac{TP+TN}{Sample size}$$

where TP, TN, FP, and FN, and represents true positive, true negative, false positive, and false negative, respectively. The accuracy was calculated from ground observations collected during April 2023.

## Results and discussion

### Classification of wheat using multi-temporal NDVI data and machine learning algorithms

**Fig. 4:**
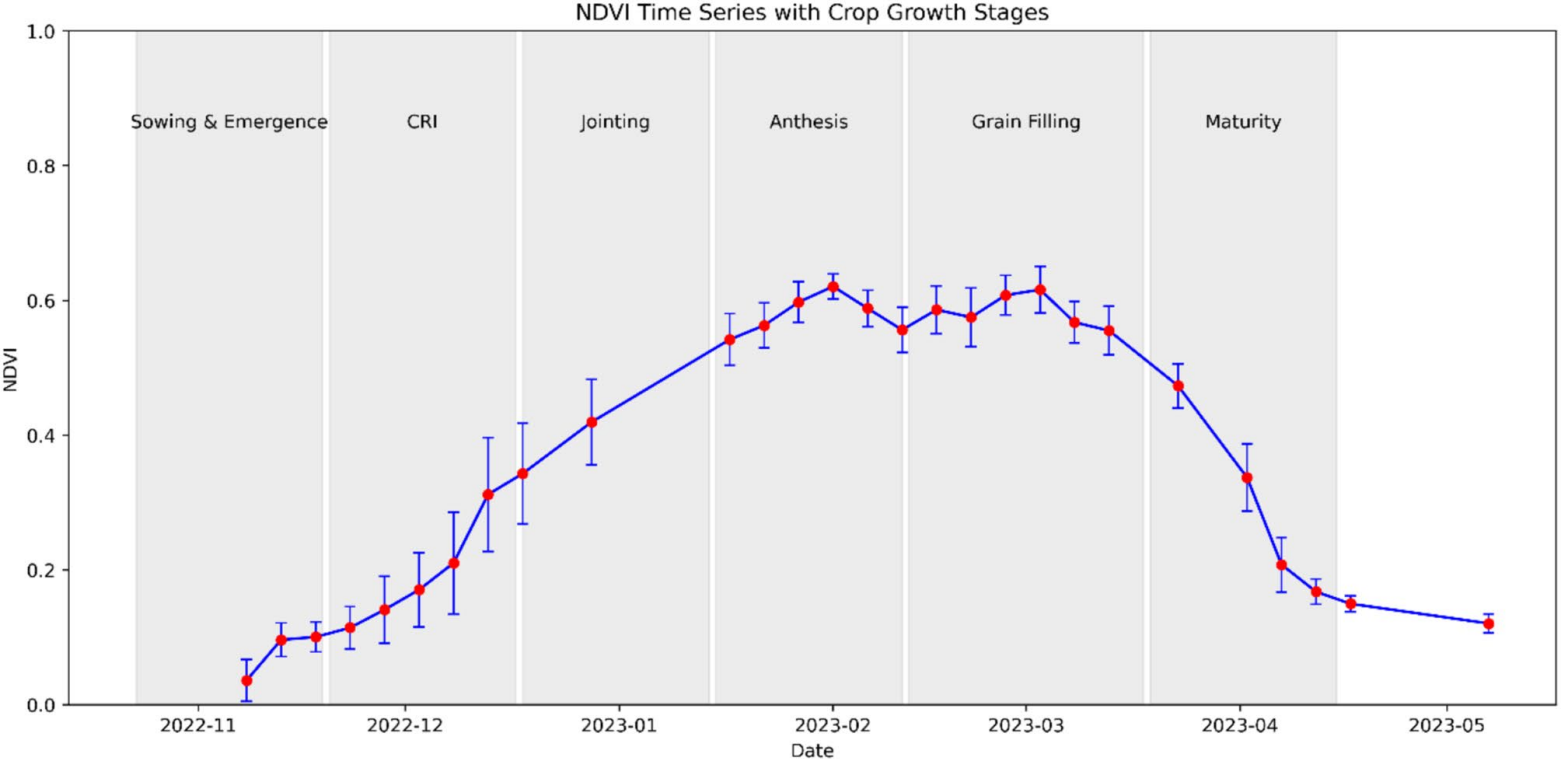
Wheat crop phenology using NDVI time series.

The temporal NDVI profiles allowed to describe the growth pattern of wheat^20,23^. The composite NDVI reflectance profile of wheat is shown in Fig. 4. The sowing period during November is characterized by a minimum NDVI value. A successive increase in NDVI values was observed during the vegetation growth from December to January. The maximum NDVI value was recorded during late January/February correspond to jointing and anthesis stages. The NDVI gradually decreased from March signifying the maturity of wheat and the chlorophyll content decreases^28^ during this period.

**Fig. 5:**
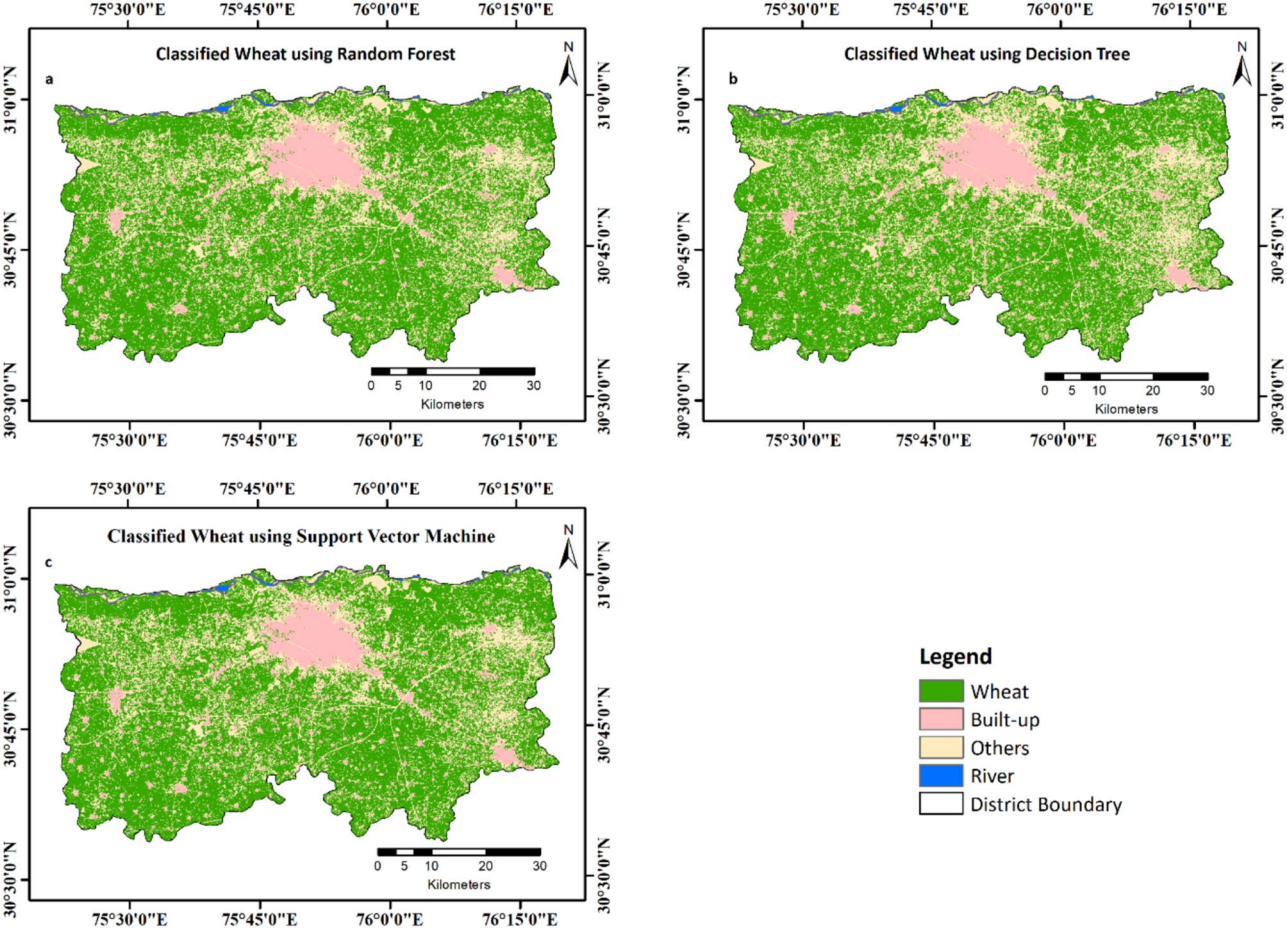
Classification of wheat area using (**a**) RF, (**b**) DT, and (**c**) SVM algorithms.

The wheat classified maps from different ML models based on the time-series NDVI data are shown in Fig. 5. Compared with DT and SVM, RF performed better in terms of overall accuracy (Table 4). The classified area under wheat was 80% of the total agricultural area of the district. However, the wheat area was overestimated by DT and SVM classifiers due to the incorrect classification of other crops and plantation as wheat. Table 4 presents classification accuracies and wheat area extracted by the respective classifiers.

**Table 4:** Performance of different ML algorithms for wheat classification.

Algorithms	Classified wheat area (% of total agricultural area)	Precision (%)	Recall (%)	F1-score	Overall accuracy (%)
RF	80.0	98.6	100	0.99	94.8
DT	86.2	97.3	99.1	0.98	93.3
SVM	91.2	95.6	98.8	0.97	88.2

### Spectral analysis of different vegetation indices for lodged and non-lodged wheat

**Fig. 6:**
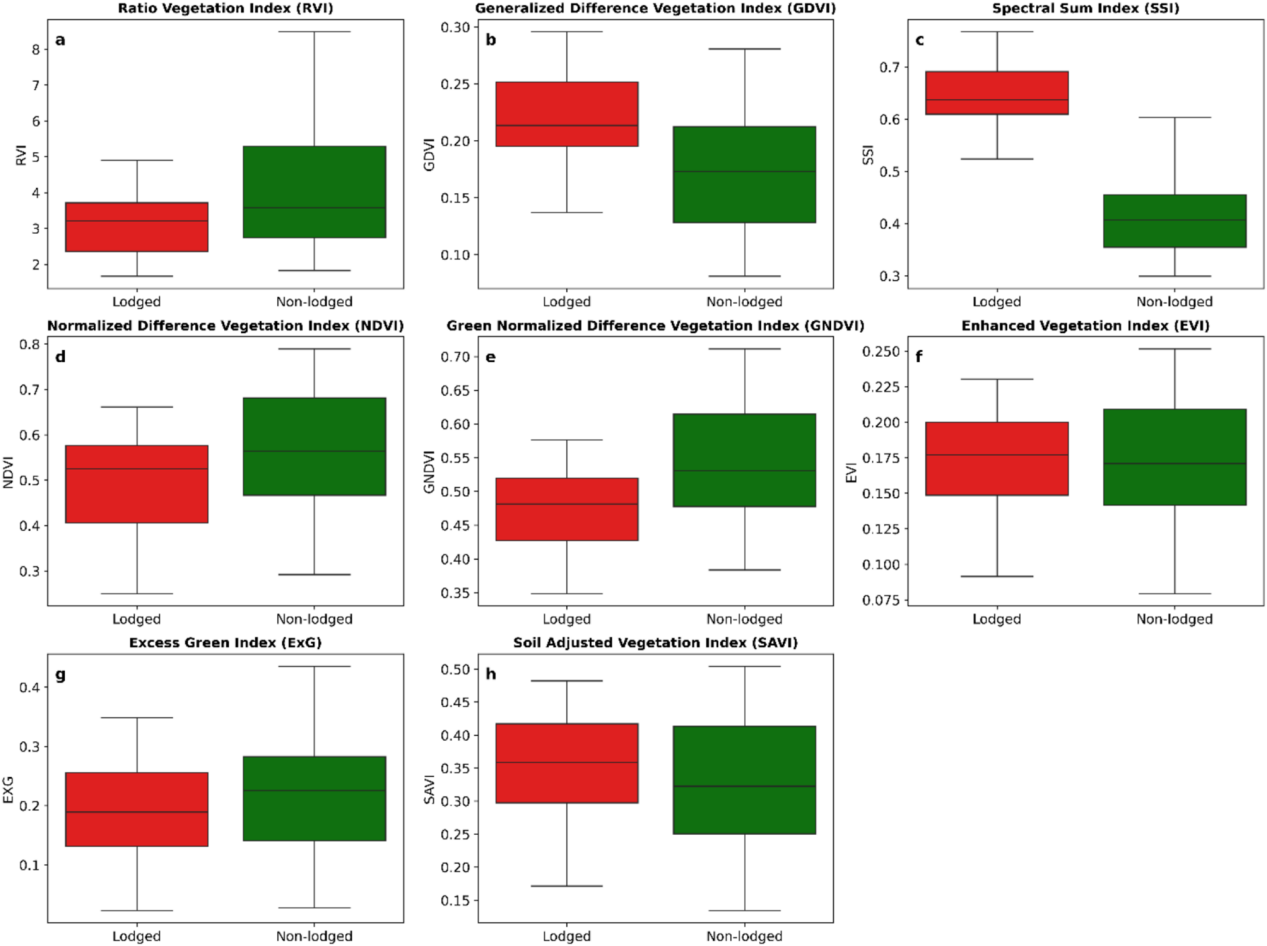
(**a**–**h**) Reflectance values of eight vegetation indices for lodged and non-lodged wheat .

**Fig. 7:**
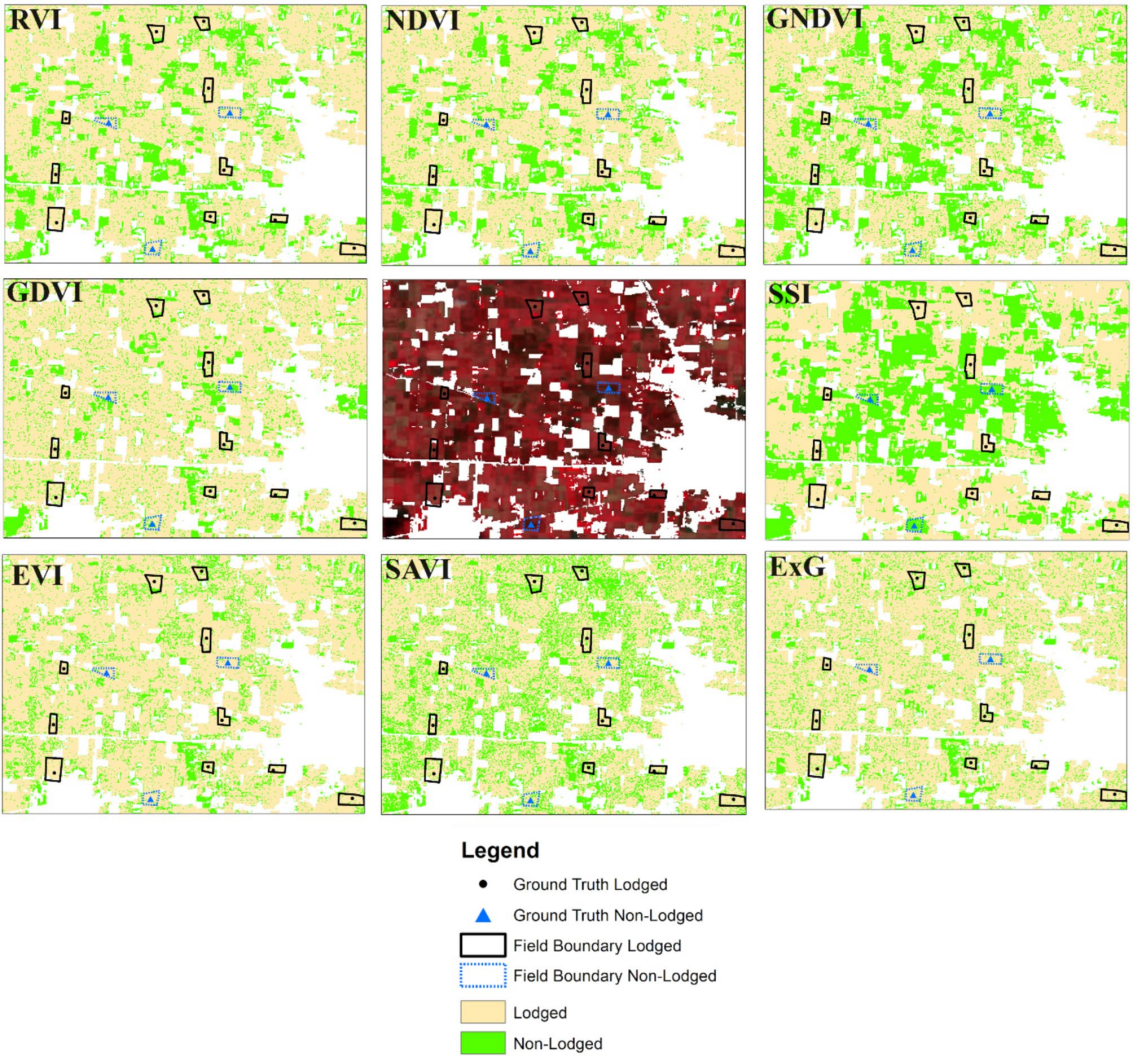
False-color composite of Sentinel-2; classified maps representing lodged and non-lodged wheat area obtained using RF with different spectral indices.

In order to study the spectral reflectance of lodged and non-lodged wheat, the ground truth samples were overlaid on the false color composite of Sentinel-2 imagery (Fig. 7). The black points, located in lodged fields were referred as lodged points, and the blue points located on non-lodged fields were referred as non-lodged points. It can be observed from the Fig. 7 that the tone of black colored field points appeared in light red and the tone of blue colored field points appeared in dark red. The tone of lodged and non-lodged points on Sentinel-2 is due to variation in reflectance, which is critical for identifying the lodged wheat. The horizontal orientation of crop stalks due to occurrence of lodging incidence increases the reflectance surface resulting in higher reflectance in visible and near-infrared bands^9^. Figure 6 visually presents the difference in the reflectance values between lodged and non-lodged wheat for different indices. SSI index, derived from summation of visible and infrared bands, has better differentiated the lodged from non-lodged wheat (Fig. 6c). It can be observed from Fig. 6d,e that there was no distinct separation between lodged and non-lodged wheat using NDVI and GNDVI indices owing to the fact^9^ that minus sign can weaken the spectral change trend as visible and near-infrared bands increase simultaneously. The mean NDVI and GNDVI of lodged wheat was lesser than the non-lodged wheat. No significant differences in reflectance values were observed across lodged and non-lodged wheat for the remaining indices.

### Influence of different vegetation indices on machine learning model performance for assessing wheat lodging

**Fig. 8:**
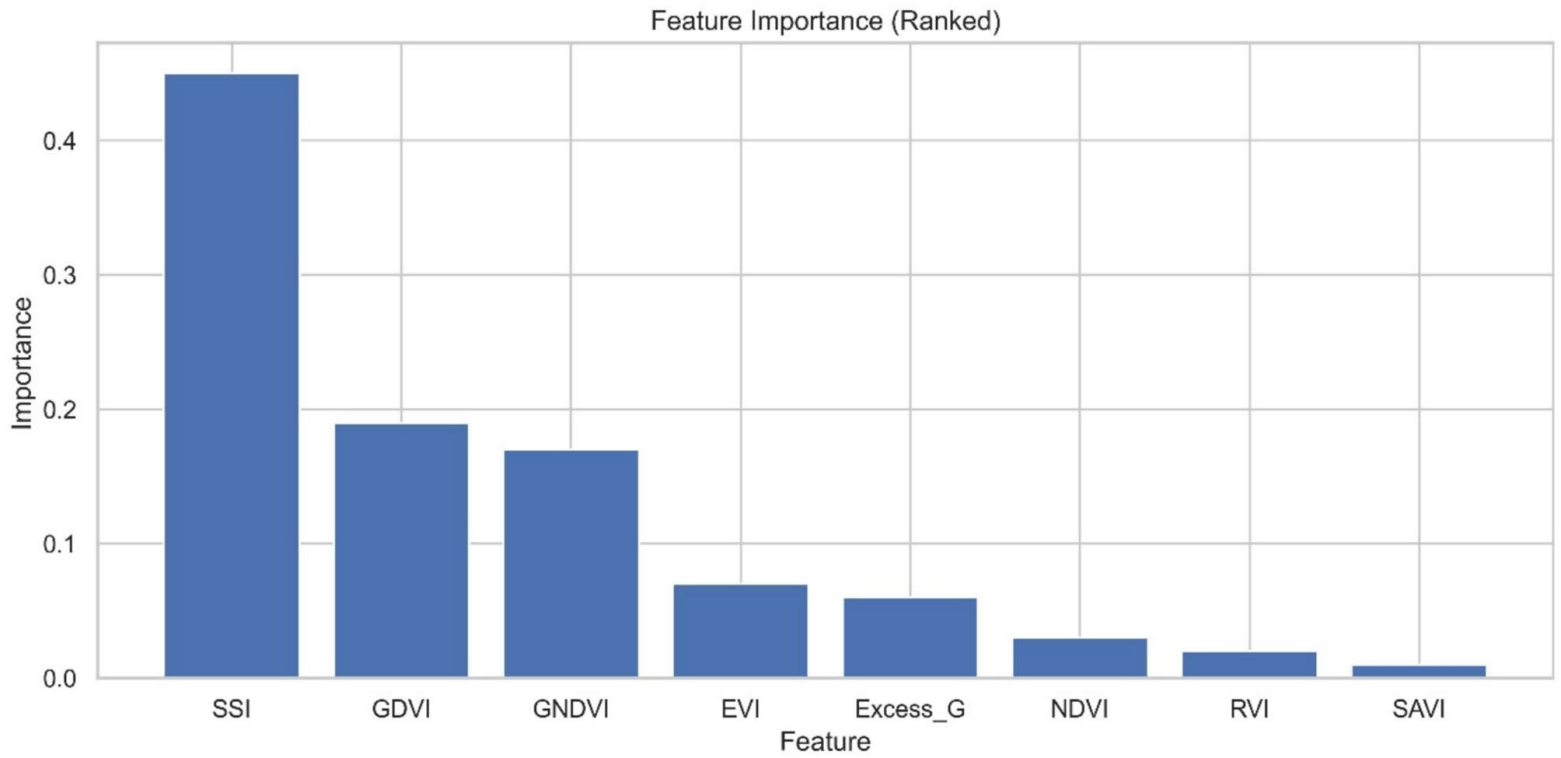
Random forest based feature importance ranking.

Initially mapping of lodged/non-lodged wheat using different spectral indices was carried out by RF algorithm and the subsets of processed images were visualized as shown in Fig. 7. The lodged wheat fields were highlighted with black boundary and non-lodged fields are highlighted with blue boundary. As illustrated from Fig. 7, RF with SSI effectively identified the lodged wheat compared to other spectral indices. From visual assessment, EVI, SAVI, and ExG indices resulted in misclassification of lodged fields. Further, Fig. 8 represents the feature importance score assessed using RF-IF approach. The features were sorted according to their importance score and the less relevant features (with importance score < 0.1) were eliminated. The SSI was identified as the most significant feature for the wheat lodging assessment followed by GDVI, and GNDVI (Fig. 8). The performance of different machine learning algorithms namely RF, DT, and SVM was evaluated with selected features based on statistical parameters (Table 5). The RF model coupled with SSI and GDVI achieved the highest overall accuracy of 89.2% (Table 5). This combination also showed the precision of 96.3% recall of 89.5% and F1-score of 0.93, for classifying wheat lodged areas (Table 6). SVM exhibited the lower overall accuracy compared with RF and DT model. The inclusion of feature selection resulted in improving the classification accuracy with RF-based models consistently outperforming DT and SVM in this analysis. The evaluation results of RF, DT, and SVM algorithms using optimal features (SSI and GDVI) for classifying non-lodged (NL) and lodged (L) wheat is given in Table 6.

**Table 5:** Performance analysis of different machine learning models using the selected features for wheat lodging assessment.

Algorithms	Selected features	Precision (%)	Recall (%)	F1-score	Overall accuracy (%)
FI-RF	SSI	87.9	97.0	0.92	85.7
FI-DT		89.6	89.6	0.89	82.0
FI-SVM		95.1	82.3	0.88	81.0
FI-RF	SSI + GDVI	96.3	89.5	0.93	89.2
FI-DT		90.0	87.8	0.88	83.8
FI-SVM		86.3	87.3	0.87	81.1
FI-RF	SSI + GDVI + GNDVI	88.3	97.0	0.92	86.2
FI-DT		90.1	93.9	0.91	85.7
FI-SVM		95.0	81.7	0.87	80.4

*FI* Feature Importance.

**Table 6:** Confusion metrics and model performance of non-lodged (NL) and lodged (L) classes of wheat using machine learning algorithms.

Algorithms with indices (SSI + GDVI)	Actual samples	NL	L	Precision (%)	Recall (%)	F1-Score	Overall accuracy (%)
RF	NL	22	9	71.0	88.0	0.79	89.2
	L	3	77	96.3	89.5	0.93	
DT	NL	21	10	67.7	72.4	0.70	83.8
	L	8	72	90.0	87.8	0.88	
SVM	NL	21	10	67.7	65.6	0.66	81.1
	L	11	69	86.3	87.3	0.87	

**Fig. 9:**
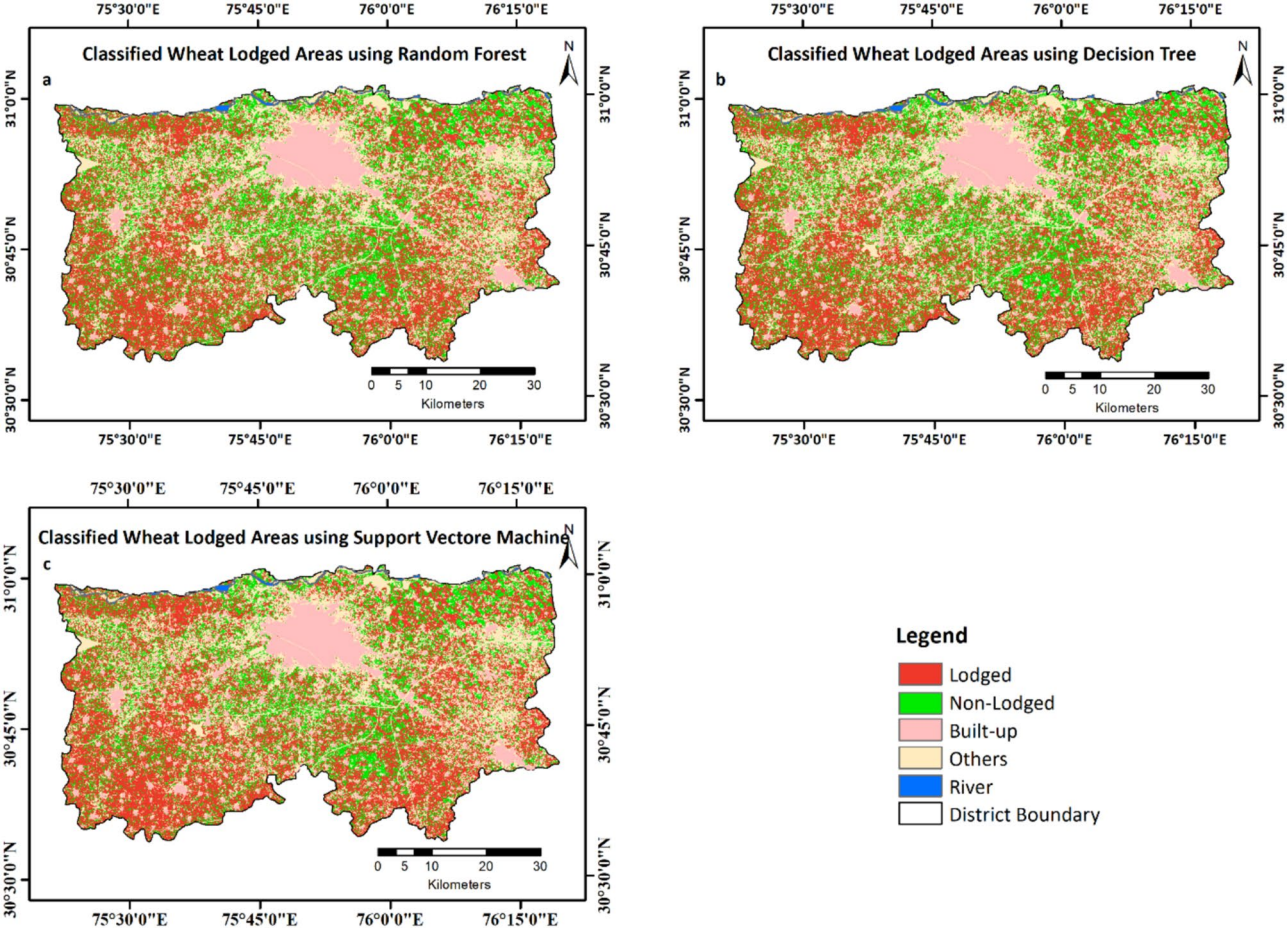
Lodged and non-lodged wheat area mapped using different ML algorithms (**a**) RF, (**b**) DT, and (**c**) SVM.

Figure 9 shows the classified maps of the lodged and non-lodged wheat area across the study region, generated using different ML models based on an optimal subset of spectral indices. The RF model identified lodging in 56.7% of the wheat area, achieving an overall accuracy of 89.2% with an overall accuracy of 89.2%. In comparison, the Decision Tree (DT) and Support Vector Machine (SVM) models mapped lodged wheat areas covering 58.0% and 61.6% of the total wheat area in Ludhiana district, respectively.

## Discussion

The present study demonstrated the potential of spectral indices for wheat lodging assessment. Wheat was classified using NDVI-time series dataset and the classifiers’ performances was evaluated using different ML algorithms. Sun et al.^29^ recommended the use of NDVI temporal information combined with RF classifier for crop classification. The present results also showed that RF with NDVI time-series exhibit good performance in mapping wheat area.

**Fig. 10:**
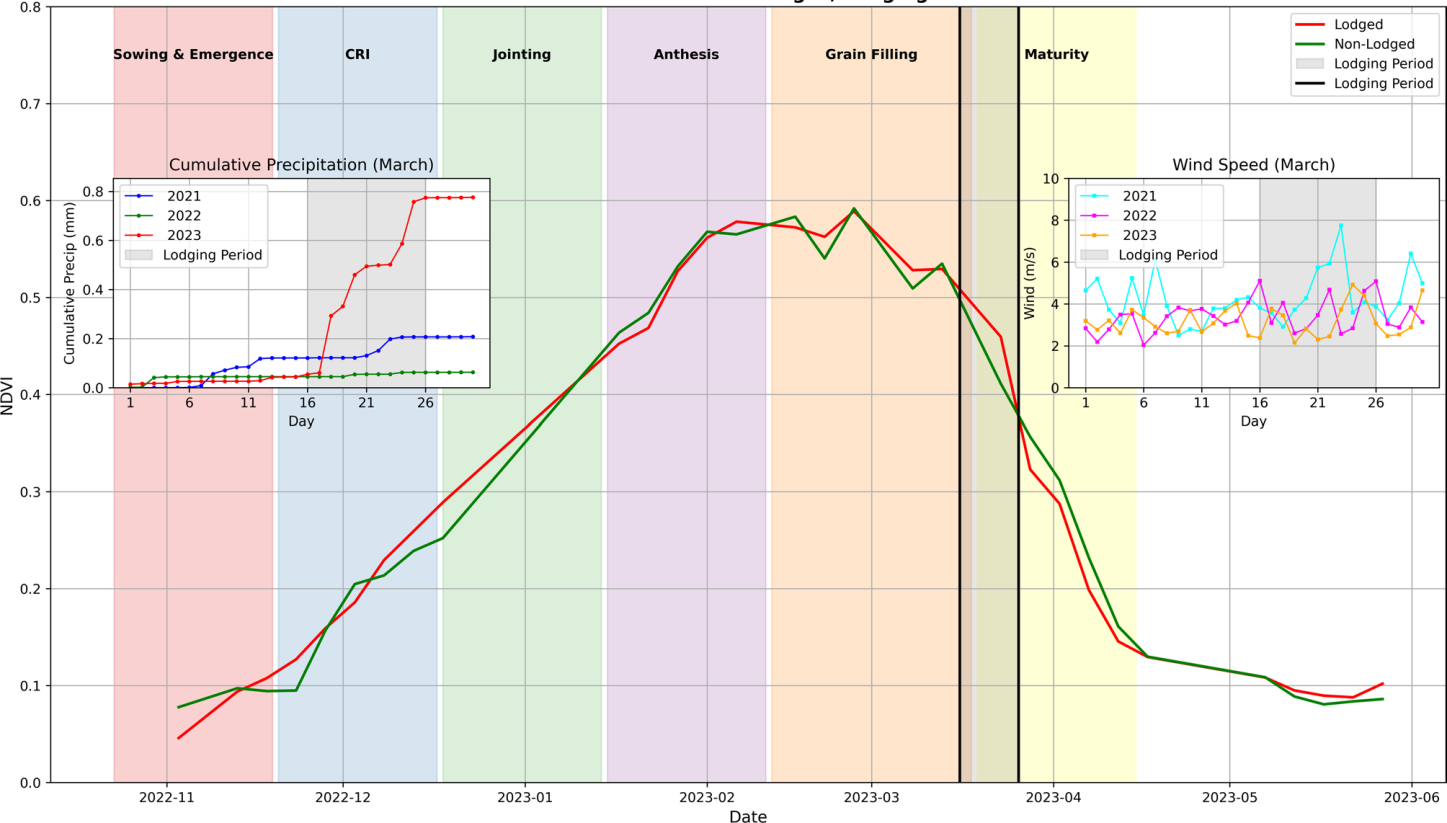
NDVI time-series curve for lodged and non-lodged wheat areas during different crop growth stages and the identified lodging period along with cumulative precipitation (top-left inset), and wind speed trends (top-right inset) during March 2023  .

In Punjab, heavy rainfall at the end of March 2023 coupled with high wind speed caused wheat lodging ^30^ (Fig. 10). Despite the lodging occurred during the maturity stage, the NDVI values showed a typical decrease as the crop transitioned into maturity. During the end of March 2023, grains were already filled before lodging took place in the Ludhiana district of Punjab. Though wheat lodging causes loss of grain yield and deterioration of wheat quality due to poor grain-filling^31^ but Mondal^32^ reported that lodging after early grain filling stage caused a smaller effect on the grain quality of wheat. The average grain yield of wheat in Ludhiana district was 5.10 t ha^−1^ during 2019–20, 4.97 t ha^−1^ during 2020–21, 4.28 t ha^−1^ during 2021–22 and 4.59 t ha^−1^ during 2022–23 (Directorate of Statistics, Government of Punjab). The trend of wheat yield over the years showed that wheat yield during 2022–23 was not affected by lodging.

Wheat lodging assessment on spatio-temporal scale is important for supporting the agricultural policies framed by government, verification of claims made by farmers applying for public subsidies, and also adopting the practices of precision agriculture. A number of studies have used vegetation indices (VIs) for lodging assessment. Tang et al. ^14^ identified NDVI as the most suitable index among EVI, RVI, and GNDVI for monitoring wheat lodging. Spectral sum index accurately distinguished lodged and non-lodged maize and was found better than NDVI^9^. Thus, the NDVI was unable to capture the structural changes of lodged crop^9^. The ExG index was used to estimate the rice lodging^33^. In the present study, the SSI index indicated an increase in spectral reflectance of lodged wheat in all bands facilitating clear discrimination from the non-lodged wheat^9^. Figure 6 shows that EVI, SAVI, and ExG did not exhibit significant changes with alterations in canopy structure after wheat lodging and thus failed to effectively assess lodging.

Several studies have compared the performance of different ML algorithms for crop lodging assessment. It has been observed that the Random Forest (RF) algorithm consistently outperforms others in terms of overall accuracy. In the present study as well, the RF algorithm accurately mapped the lodged fields, achieving the highest overall accuracy of 85.7%–89.2%, outperforming other ML algorithms and supporting the findings of previous studies^18,29^. The feature importance is important for building the ML models and a random forest-based feature importance approach resulted in identifying the most important features (indices) for classifying the lodged wheat. The RF algorithm ranked the SSI index as the most important feature followed by GDVI for assessing wheat lodging. RF model coupled with SSI and GDVI indices achieved the highest overall accuracy and outperformed other model-index combinations. The lodging assessment results confirm the capability of proposed approach in detecting the wheat lodging area.

The current study evaluated the performance of spectral indices to assess the wheat lodging on spatial scale. One of the key limitations of this study is the reliance on optical sentinel-2 imagery, which is often limited by cloud cover and adverse weather conditions leading to gaps in temporal coverage. Additionally, the spatial resolution of 10 m may result in mixed pixels particularly in areas with small lodged patches, thereby affecting the accuracy of classification. The inclusion of microwave data, which can provide consistent observations regardless of weather, can improve the detection of lodged wheat^3^. These factors underscore the need for integrating higher-resolution and multi-source data to enhance the reliability of wheat lodging detection.

## Conclusions

The present study has explored the potential of time-series multispectral satellite data, integrated with machine learning algorithms for wheat lodging assessment over agricultural fields. Addressing the limitations of previous field-specific studies dependent on UAV imagery, this research systematically evaluated the spectral behavior of eight vegetation indices using high-resolution Sentinel-2 data for large-scale mapping. The integration of SSI and GDVI with the RF model is useful for assessing wheat lodging which was mapped with an overall accuracy of 89.2%. These results demonstrated that the proposed methodology enables robust, scalable, and precise extraction of wheat lodging events using satellite-based monitoring. The comprehensive analysis of spectral indices and the identification of optimal indices for lodging detection may serve as reference for future wheat lodging studies. The proposed approach offers farmers a rapid and cost-effective way to identify the lodged areas, supporting timely management decisions to minimize yield losses. The future research could explore the application of deep learning (DL) algorithms to capture complex spatial and temporal patterns associated with wheat lodging for further improving the model robustness and accuracy.

## Data Availability

The datasets utilized and/or analyzed in the present study can be obtained from the corresponding author upon reasonable request.
